# Resilience displays similar associative patterns with academic achievement regardless of the personality and mental health profile of future teachers

**DOI:** 10.1186/s40359-025-03697-7

**Published:** 2025-12-11

**Authors:** Jana Kvintová, Lucia Lacková, Hongyang Liu, Lucie Váchová, Milon Potmesil, Veronika Růžičková, Kamil Kopecký, Vojtech Regec

**Affiliations:** 1 Department of Psychology and Abnormal Psychology, Faculty of Education, Palacky University in Olomouc, Czech Republic Žižkovo nám. 5, CZ 779 00 Olomouc,; 2 Institute of Special Education Studies, Faculty of Education, Palacky University in Olomouc, Žižkovo nám. 5, CZ 77900 Olomouc, Czech Republic; 3 Department of Czech Language and Literature, Faculty of Education, Palacky University in Olomouc, Žižkovo nám. 5, CZ 779 00 Olomouc, Czech Republic

**Keywords:** Resilience, Academic achievement, Teacher training, Personality traits, Mental health

## Abstract

**Aim:**

Given the demanding nature of teacher education, understanding how resilience relates to academic success and social adaptation is crucial. The aim of this study was to examine the relationship between resilience and academic achievement and to determine whether this relationship varies based on the personality/mental health profile of future teachers.

**Method:**

A total of 793 university students enrolled in teacher training programs participated in this study. Data were collected via validated questionnaires measuring resilience (CD-RISC-25) with 5-factor solution including (1) Positive acceptance of change and secure relationships, (2) Trust in one’s instincts, tolerance of negative affect, and strengthening effects of stress, (3) Personal competence, high standards, and tenacity, (4) Control, and (5) Spiritual influences (entered as explanatory or predictor variables in regression models); academic achievement (AAQ) with (1) Study performance, (2) Coping with study demands and (3) Social adaptation domains (treated as dependent variables); personality traits (TIPI) covering Big Five traits (as input for cluster analysis); and mental health indicators (PHQ-4) including depression and anxiety (as input for cluster analysis). Analyses were associative and exploratory; terms such as ‘predictor’ denote statistical prediction, not causation. First, correlation and regression analyses were conducted to determine the overall association between resilience and academic achievement. Next, these associations were tested in groups with different personality/mental health profiles (based on cluster analysis).

**Results:**

The findings of the whole sample analysis revealed that resilience was significantly associated with coping with study demands and social adaptation, accounting for ~ 21% and 30% of the variance in those outcomes, respectively. In particular, Personal Competence, High Standards, and Tenacity (PCHST) and Positive Acceptance of Change and Secure Relationships (PACSR) were found to show the strongest association. In contrast, resilience did not manifest direct association with academic performance. Subsequent subgroup analysis showed that relationship between resilience and academic achievement remained consistent across different personality or mental health profiles, suggesting relevance across personality/mental-health profiles.

**Conclusion:**

The cross-sectional findings indicate that resilience is significantly related to the academic achievement of future teachers, and this relationship is independent of their personality structure and mental health issues. These findings underscore the potential importance of resilience in enhancing coping mechanisms and social integration among future teachers. Teacher training programs may consider incorporating resilience-building strategies to support students in managing academic stress and fostering professional preparedness.

**Supplementary Information:**

The online version contains supplementary material available at 10.1186/s40359-025-03697-7.

## Introduction

The teaching profession demands not only intellectual rigor but also significant emotional resilience and adaptability. Students in teacher training programs routinely encounter high workloads, including intensive coursework, lesson planning, and frequent assessments, all of which require effective time management and sustained motivation. Additionally, the transition from student to teacher involves practicum placements, where pre-service teachers must manage real classrooms, handle diverse student behaviors, and navigate complex relationships with mentors and peers. These experiences are often accompanied by emotional labor—balancing personal well-being with the responsibility of supporting students’ academic and emotional needs—as well as adapting to evolving educational policies, new technologies, and multicultural classroom dynamics [1].

Given their central role in shaping future generations, it is critical to understand the factors that contribute to the academic and personal success of teacher candidates. Resilience has emerged as a pivotal protective factor, helping pre-service teachers manage academic stress, maintain motivation, and persist through setbacks during coursework and practicum experiences [2, 3]. Research suggests that resilience enhances professional identity formation, reduces burnout, and supports long-term career commitment in teacher education [4]. However, the influence of resilience on academic outcomes is not uniform. Individual differences such as personality traits—especially conscientiousness, neuroticism, and extraversion—as well as levels of anxiety and depression, have been shown to moderate the relationship between resilience and academic performance [5, 6].

Resilience, broadly defined as the ability to recover from adversity and maintain adaptive functioning [7], or the capacity to be competent despite a stressful situation [8], is a dynamic construct that encompasses emotional, cognitive, and social resources. Gu and Day (2013) underscore that resilience is not a fixed trait but rather a developmental process influenced by personal and environmental factors [9]. This perspective aligns with Bronfenbrenner’s ecological systems theory, which suggests that resilience is shaped by the interaction of individual characteristics and surrounding systems [10]. Masten (2014) further emphasizes the “ordinary magic” of resilience, highlighting its universal applicability across populations and contexts [11]. More recently, Masten (2023) conceptualizesresilience as a multisystem adaptive capacity that emerges through the coordination of resources across individual, relational, and contextual domains, underscoring its universal applicability across populations and contexts [12]. In educational settings, the issue of resilience is a subject of increasing relevance [13].

Resilient students tend to engage more deeply in class, persevere through setbacks, and earn higher grades [14, 15]. Engagement thrives when students feel intrinsically motivated, supported by teachers, and confident in their abilities. Perseverance—essentially “grit”—is fostered by conscientiousness and clear goals, while achievement benefits from emotion regulation and effective coping.

For pre-service teachers, resilience is equally pivotal. More resilient candidates report less stress and burnout, greater classroom-management confidence, and stronger intent to remain in the profession [3, 4]. Key mechanisms include better emotion regulation, problem-solving, and cognitive reappraisal [16], plus higher self-efficacy and goal orientation [17, 18]. By buffering emotional exhaustion, resilience ultimately strengthens commitment to teaching [3, 19].

Academic achievement at university encompasses various dimensions, primarily focusing on the performance outcomes of students in their academic pursuits (typically GPA). However, other components such as coping with academic demands and social adaptation are likewise integral components of academic achievement [20]. Moreover it reflects a combination of cognitive ability, effort, and contextual influences [21]. The relationship between resilience and academic achievement is well-documented, when resilient individuals demonstrating higher levels of perseverance, problem-solving skills, and adaptability [22] and tend to achieve better academic results, as resilience helps them manage stress and overcome obstacles in their learning environment [23]. While resilience is a key factor in academic achievement, it is essential to consider the interaction of other variables such as personality traits and mental health, as these elements can either support or hinder a student’s learning journey.

Personality traits, particularly those outlined in the Five-Factor Model [24], have been consistently linked to academic achievement. High levels of conscientiousness and openness to experience are predictive of greater academic engagement, goal persistence, and self-regulated learning, all of which contribute to academic success [25, 26]. In contrast, high neuroticism and low extraversion have been associated with academic anxiety, burnout, and lower performance during practicum and coursework [27].

Also, these traits significantly influence individual responses to stress and capacity for resilience. For instance, resilience is negatively correlated with neuroticism, while the other Big Five dimensions are positively associated with resiliency [28], similarly, all Big Five factors were positively related to resilience, with Impersonal Orientation (low self-determination) mediating the relationship between extraversion, agreeableness, conscientiousness, and openness on the one hand and resilience on the other [29]. Finally, there was a positive relationship between student resilience and academic progress. Furthermore, student resilience was associated with four protective personality factors, namely openness, conscientiousness, extraversion, and emotional stability [30].

Mental-health conditions—especially depression and anxiety—impair attention, working memory, and motivation, heighten procrastination and disengagement, and raise the risk of lower GPA, course non-completion, and academic withdrawal [31–34]. Such symptoms can also erode the protective value of resilience by limiting students’ use of adaptive coping strategies [35]. Within this dynamic, personality traits and mental health act as moderators of the resilience–achievement link: the buffering effect of resilience is strongest for students low in neuroticism and experiencing fewer depressive or anxious symptoms [36, 37].

Studies reveal that anxiety and depression impair attention, working memory, and executive functioning, which are critical for effective learning and performance in higher education [38–40]. They also disrupt motivation, contribute to difficulties in decision-making, and increase tendencies toward procrastination and academic disengagement. Emotional distress is frequently linked to sleep disturbances, which further exacerbate cognitive impairment and academic difficulties [41, 42].

Effective coping skills and high resilience partly offset these harms; students who score higher on resilience manage stress better and maintain performance, confirming its *mediating* role between personality and anxiety [5, 43–45]. Conversely, severe or chronic anxiety and depression can undermine resilience and predict poorer outcomes, including test anxiety, disengagement, and early dropout—particularly when stress remains unmanaged [6, 46–48]. The Transactional Model of Stress and Coping and Resilience Theory both underscore that withstanding academic pressures depends on the interplay of coping behaviours, personality traits, and environmental supports—an interaction that is especially salient for pre-service teachers facing heavy coursework, practicum stress, and identity formation [49, 50].Despite growing interest in academic resilience, little research has comprehensively examined how it interacts with both personality traits and mental health among pre-service teachers. This population is uniquely positioned at a transitional stage, balancing academic responsibilities and the developmental tasks of becoming educators. By integrating these theoretical perspectives and recent findings, this study aims to contribute to a deeper understanding of how resilience, personality, and mental health intersect to shape the academic trajectories of future teachers. Specifically, the goal of this study was to examine the relationship between resilience and academic achievement and to determine whether this relationship varies based on the personality/mental health profile of future teachers. These insights might have significant implications for the design of teacher education programs, emphasizing the importance of fostering resilience and supporting mental health to enhance academic success.

## Methods

### Study design and sample

This study employed a cross-sectional quantitative design to investigate the relationship between resilience, academic achievement, personality traits, and mental health among university students preparing for teaching careers. All 1,603 students enrolled in psychology-related disciplines within teacher-preparation programs at the Faculty of Education, Palacký University in Olomouc (Czech Republic) were invited to participate. Of these, 793 completed the survey, giving a response rate of 49.47%; the remaining 810 students did not respond. All submitted questionnaires were valid and included in the analysis, as none were excluded due to incomplete or inconsistent responses because all items in the online questionnaire were mandatory.

The data collection took place in a controlled classroom environment between 14/2/2022 to 31/05/2022 and 13/02/2023 to 31/05/2023. Participants were invited to complete the questionnaire using their personal mobile phones during class time, ensuring a structured and uniform setting for data collection.

The sample consisted of 793 university students (future teachers) with mean age ± SD: 23.7 ± 6.4. Of these, 666 (84%) were females and 127 (16%) were males. The students were most frequently attending the second (*N* = 485, 61.2%) and first (*N* = 189, 23.8%) year of study. The main characteristics of the population were represented in a proportion corresponding to the sample, rendering the sample representative.

### Measures

Resilience was measured using Connor-Davidson Resilience Scale (CD-RISC-25) [51]. This 25-item scale assesses five domains of resilience on a 5-point Likert scale (0 = not true at all’, 4=’true nearly all of the time’): (1) Positive acceptance of change and secure relationships (PACSR), (2) Trust in one’s instincts, tolerance of negative affect, and strengthening effects of stress (TITNA), (3) Personal competence, high standards, and tenacity (PCHST), (4) Control (CNT), and (5) Spiritual influences (SI). Given that previous studies have reported different factor structures for this instrument, we verified whether the original factor structure was appropriate for our population. A series of factor analyses confirmed that the selected 5-factor solution was indeed the most accurate (Supplementary Table S1) with sufficiently independent factors (inter-factor correlation ranging from 0.19 to 0.53). The questionnaire showed very good overall reliability of ω = 0.91 (with subscale reliabilities ranging from 0.6 to 0.77). Permission to use the CD-RISC-25 was obtained from the author Jonathan R. Davidson, M.D.

Academic achievement was assessed using Academic Achievement Questionnaire (AAQ) [52]. This new 9-item scale measures overall academic achievement and its three dimensions using 6-point Likert scale (1=’Not at all’, 5=’Without problem’): study performance, coping with study demands and social adaptation. Reliability (McDonald’s omega) proved to be good (ω_total_ = 0.77, ω_st.perf_. = 0.76, ω_st.dem_. = 0.79, ω_soc.adapt_. = 0.59).

Depressive and anxiety symptoms were assessed using the 4-item version of the Patient Health Questionnaire (PHQ-4) [53]. This short tool with 4-point Likert scale (0=’ Not at all’, 3=’Nearly every day’) is designed to screen for the presence of depressive and anxiety symptoms. Higher scores indicate greater presence of symptoms. Despite its shortness, it has shown good psychometric properties. Reliability in this study was ω_depr_. = 0.77, ω_anx_. = 0.85.

Personality traits were assessed using Ten Item Personality Measure (TIPI) [54]. This short 10-item questionnaire assesses five personality traits based on the Big Five using 7-point Likert scale (1=’Strongly disagree’, 7=’Strongly agree’): Extraversion, Emotional stability (Neuroticism), Agreeableness, Conscientiousness, and Openness to experience. Despite being short, the questionnaire has proved its usefulness and validity when compared to longer scales [54, 55], and there are also studies that show that this questionnaire is applicable to individuals in different countries [56–59]. Reliability was acceptable for most traits with ω_ext_. = 0.67, ω_aggr_. = 0.21, ω_consc_. = 0.59, ω_emst_. = 0.59, and ω_open_. = 0.45. Lower omega values for extraversion and openness can be attributed to the TIPI design and generally lower reliability of scales with a small number of items [60, 61].

### Statistical analysis

Reliability of tools was examined using McDonald’s omega. A series of factor analyses were used to assess the goodness-of-fit of the factor structure of CD-RISC-25 questionnaires with different numbers of factors (tested range 1–5 factors, oblimin rotation). Goodness-of-fit was evaluated using several indicators: Root Mean Square Residual (RSMR, should be < 0.08), Root Mean Square Error of Approximation (RSMEA, should be < 0.05), Tucker Lewis index (TLI, should be > 0.90), and Comparative Fit Index (CFI, should be > 0.90). Preliminary presence of relationships between resilience and academic achievement was verified by Spearman correlation analysis and multiple regression analysis. Stratification of students into subgroups based on their personality/mental health profile was performed using k-means clustering utilizing personality traits and mental health issues, namely depressive symptoms and anxiety. A subsequent series of multiple regression analyses within each cluster examined the effect of resilience factors on academic performance conditional on cluster membership. Finally, to assess the role of personality/mental health profiles of future teachers in the relationship between resilience and academic achievement, we examined differences in regression models between clusters of participants for each dependent variable (academic achievement factor) using two indicators: model efficiency and structure. The presence of differences in the efficiency of the models was verified using the Z test for differences in the square root of adj. R^2^ values (adapted with Fisher’s r-to-Z transformation). A series of three pairwise comparison of the structure of the regression weights of the models from individual cluster pairs was conducted by applying the model derived from the one cluster to the data from the second cluster and comparing the resulting “crossed” R² with the “direct” R² originally obtained from this cluster by Steiger’s Z test.

All statistical analyses were performed as two-tailed and all the *P* < 0.05 were considered statistically significant. Data analysis and visualizations were performed in RStudio (v.2024.04.2 build 764, with R environment v.4.4.0).

## Results

**Fig. 1:**
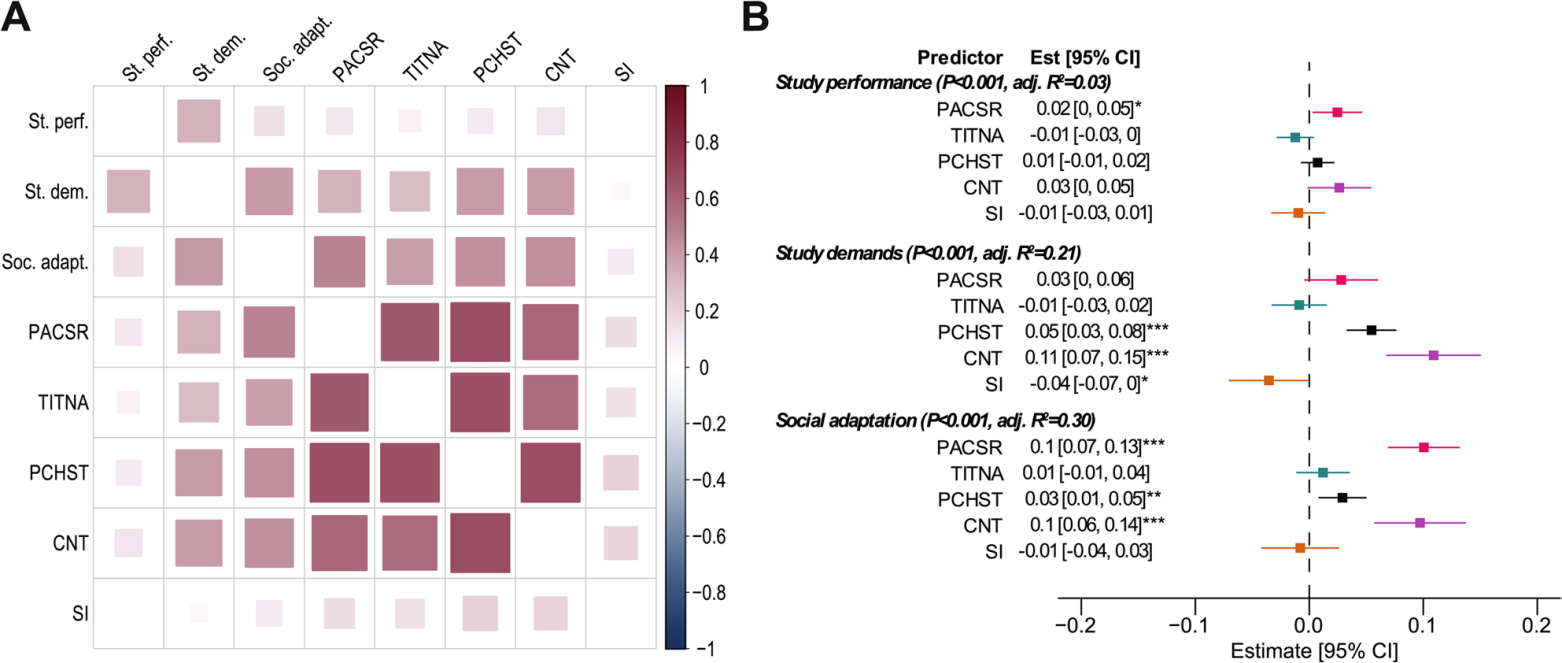
A preliminary analysis of the presence of relationships between resilience and academic achievement. (**A**) The heatmap shows the individual correlations between the resilience domains and the academic achievement subscales. (**B**) Forest plot shows the regression coefficients indicating the effect of resilience domains on each academic achievement subscale. Brackets after the subscale name report the overall significance of the regression model and the amount of explained variance of the dependent variable). Asterisks indicate the level of statistical significance (*P<0.05, **P<0.01, ***P<0.001). PACSR: Positive acceptance of change and secure relationships, TITNA: Trust in one's instincts, tolerance of negative affect, and strengthening effects of stress, PCHST: Personal competence, high standards, and tenacity, CNT: Control, SI: Spiritual influences

First, we verified the existence of relationships between the domains of resilience and academic achievement. Both correlation analysis (Fig. 1 A, Supplementary Table S2) and multiple regression analysis (Fig. 1B, Supplementary Table S3) confirmed that there is a significant association of resilience on academic achievement.

The regression model for study performance was significant (F [5, 787] = 5.22, *p* < 0.001), accounting for 3% of the variance (adjusted R^2^ = 0.03). Among the resilience domains, Personal Competence, High Standards, and Tenacity (PCHST) showed a positive but marginal effect on study performance (R^2^ = 0.01, *p* = 0.331), while Control (CNT) exhibited a borderline significant positive association (R^2^ = 0.03, *p* = 0.065). Other domains, such as Trust in One’s Instincts (TITNA) and Spiritual Influences (SI), did not demonstrate significant contributions to study performance, indicating that resilience may have limited direct influence on this outcome.

For coping with study demands, the regression model was highly significant (F[5, 787] = 43.26, *p* < 0.001), explaining 21% of the variance (adjusted R^2^ = 0.21). Control (CNT) emerged as the strongest unique association of coping abilities (R^2^ = 0.11, *p* < 0.001), followed by Personal Competence (PCHST) (R^2^ = 0.05, *p* < 0.001). Additionally, Positive Acceptance of Change and Secure Relationships (PACSR) exhibited a marginally significant effect on coping (R^2^ = 0.03, *p* = 0.090). These findings highlight the critical role of self-regulation and perseverance in managing academic challenges effectively.

The regression model for social adaptation was the strongest among the three subscales, achieving significance (F[5, 787] = 69.95, *p* < 0.001) and explaining 30% of the variance (adjusted R^2^ = 0.30). Positive Acceptance of Change and Secure Relationships (PACSR) had the most pronounced effect (R^2^ = 0.10, *p* < 0.001), emphasizing the importance of adaptability and social connections. Both Control (CNT) (R^2^ = 0.07, *p* < 0.001) and Personal Competence (PCHST) (R^2^ = 0.03, *p* = 0.006) also contributed significantly to social adaptation, reinforcing the role of resilience in fostering interpersonal success within academic environments.

**Fig. 2:**
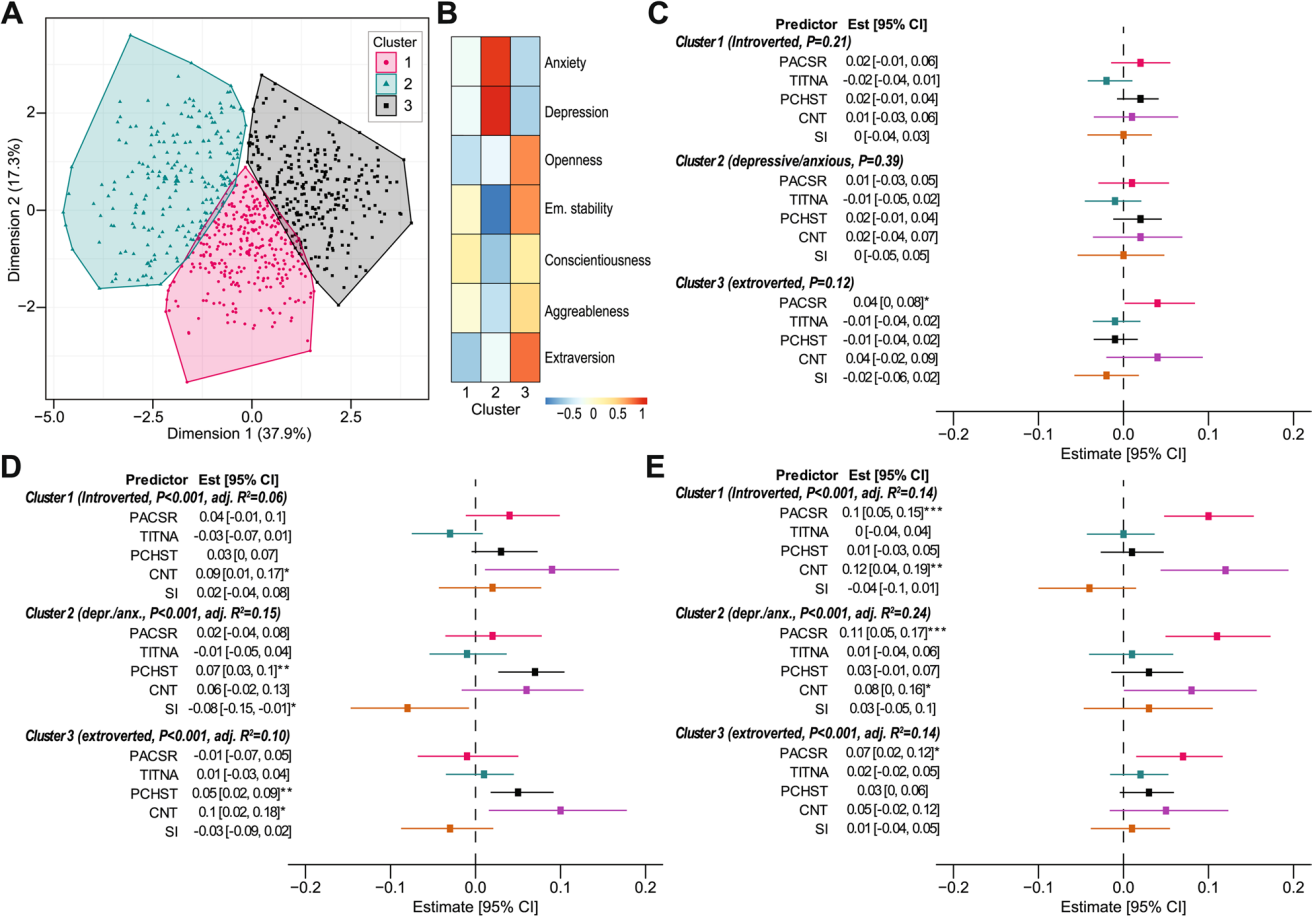
Cluster-specific effects of resilience domains on academic achievement. (**A**) Cluster plot indicates the distribution of participants into groups based on k-means clustering. (**B**) Heatmap showing standardized mean values of personality and mental health characteristics of participants in individual clusters. (**C**-**E**) Forest plots depict regression coefficients indicating the effect of resilience domains on study performance (**C**), coping with study demands (**D**), and social adaptation (**E**) across individual clusters. Brackets after the subscale name report the overall significance of the regression model and the amount of explained variance of the dependent variable). Asterisks indicate the level of statistical significance (*P<0.05, **P<0.01, ***P<0.001). PACSR: Positive acceptance of change and secure relationships, TITNA: Trust in one's instincts, tolerance of negative affect, and strengthening effects of stress, PCHST: Personal competence, high standards, and tenacity, CNT: Control, SI: Spiritual influences

Subsequently, we explored whether the observed effect of resilience on academic achievement was dependent on other variables, namely personality traits and mental health issues. We therefore divided participants into three groups using k-means clustering (Fig. 2 A). These groups were found to comprise introverted (cluster 1) and extroverted (cluster 3) individuals with low presence of mental health issues, and individuals with high presence of depressive symptoms and anxiety and reduced emotional stability (cluster 2) (Fig. 2B, Supplementary Table S4).

Regression analyses revealed no significant effects of resilience domains on study performance within any cluster. Across all clusters, the models were not statistically significant (Cluster 1: F[5, 289] = 1.45, *p* = 0.207; Cluster 2: F[5, 209] = 1.05, *p* = 0.387; Cluster 3: F[5, 277] = 1.78, *p* = 0.116), and the adjusted R² values were minimal (≤ 0.01). These results indicate a limited direct association between resilience and study performance, regardless of personality traits or mental health characteristics (Fig. 2 C, Supplementary Table S5).

Significant overall models were observed for coping with study demands in all clusters (Cluster 1: F [5, 289] = 5.06, *p* < 0.001, adj. R² = 0.06; Cluster 2: F[5, 209] = 8.35, *p* < 0.001, adj. R² = 0.15; Cluster 3: F[5, 277] = 7.31, *p* < 0.001, adj. R² = 0.10). Control (CNT) emerged as a significant predictor in Cluster 1 (β = 0.09, *p* = 0.025), while Personal Competence (PCHST) was the strongest predictor in Cluster 2 (β = 0.07, *p* = 0.001) and Cluster 3 (β = 0.05, *p* = 0.024). These results highlight slight variations in resilience’s role in coping mechanisms across clusters (Fig. 2D, Supplementary Table S6).

Resilience significantly associated with social adaptation in all clusters, with overall models yielding the highest explanatory power (Cluster 1: F[5, 289] = 10.53, *p* < 0.001, adj. R² = 0.14; Cluster 2: F[5, 209] = 14.67, *p* < 0.001, adj. R² = 0.24; Cluster 3: F[5, 277] = 10.35, *p* < 0.001, adj. R² = 0.14). Across clusters, Positive Acceptance of Change and Secure Relationships (PACSR) had significant positive effects (Cluster 1: β = 0.10, *p* < 0.001; Cluster 2: β = 0.11, *p* < 0.001; Cluster 3: β = 0.07, *p* = 0.011), demonstrating its importance in fostering adaptability and social connections (Fig. 2E, Supplementary Table S7). Additionally, Control (CNT) showed a significant association with social adaptation in introverted Cluster 1 (β = 0.12, *p* = 0.002) and marginally in depressive/anxious Cluster 3 (β = 0.08, *p* = 0.049), while it was insignificant in extroverted Cluster 2.

Tests for differences in model efficiency and structure revealed no significant differences across clusters for any academic achievement subscale (Supplementary Tables S8, S9). This consistency underscores the universal applicability of resilience across diverse personality traits and mental health profiles.

## Discussion

The results revealed that specific resilience domains are significantly associated with coping with study demands and social adaptation. Notably, the Personal Competence, High Standards, and Tenacity (PCHST) facet showed only a minor, non-significant trend with study performance, reinforcing that resilience alone has limited direct effect on grade-related outcomes, while trust in one’s instincts and tolerance of negative effect (TITNA) positively correlated with better coping, though its unique contribution was not prominent when accounting for other resilience factors. Positive acceptance of change and secure relationships (PACSR) played a critical role in social adaptation. The results showed that resilience was related to better coping with study demands and social adaptation in all clusters, though the specific effects varied slightly. However, resilience had no direct impact on academic performance, irrespective of personality or mental health characteristics. The consistency in the efficiency and structure of regression models across clusters is consistent with a broad pattern whereby resilience is associated with non-grade aspects of academic achievement.

The findings align with prior research on teacher education students that underscore the pivotal role of resilience in navigating the demands of professional training. For example, Tait (2008) highlighted that resilience predicts long-term success and professional engagement among pre-service teachers, suggesting that resilience serves as a protective factor in sustaining motivation and managing academic and emotional stress [4]. Likewise, Brunetti (2006) emphasized that resilient student-teachers demonstrate higher levels of personal commitment, reflective capacity, and persistence during challenging academic periods [62]. These observations are consistent with the current study, where resilience dimensions such as personal competence and adaptability significantly contributed to non-grade aspects of academic achievement like coping and social adaptation.

Other studies focusing specifically on teacher trainees reinforce these findings. Hong (2012) found that preservice teachers with stronger resilience profiles were more likely to persist in their education programs despite academic and emotional challenges [3]. Similarly, Mansfield et al. (2016) noted that resilience not only supports academic success but also enhances professional identity formation and long-term teaching efficacy [19]. These insights echo the present study’s conclusion that resilience operates as a universal psychological asset, transcending individual personality or mental health differences, and plays a crucial role in preparing future teachers for both academic and professional pressures.

Distinctively, this study employed cluster analysis to reveal the uniform impact of resilience across diverse subgroups, contributing novel insights into how resilience functions among future teachers with varying personality traits and mental health profiles. This finding echoes the work of Beltman et al. (2011), who emphasized that resilience in preservice teachers is shaped by both personal resources and contextual challenges throughout their training [2]. Moreover, García-Martínez et al. (2022) demonstrated that resilience consistently mediated the relationship between emotional intelligence and mental health across a large sample of pre-service teachers, regardless of personality traits [63]. These parallels underscore the potential of resilience-building as a universal strategy in teacher education.

Personal competence demonstrated a strong positive relationship with study performance. This finding aligns with Bandura’s Self-Efficacy Theory, which posits that belief in one’s capabilities influences motivation and academic behaviors [64]. Students with high personal competence, high standards, and tenacity (PCHST) exhibit goal-oriented behavior, persistence, and effective self-regulation, enabling better performance in structured academic environments [65]. This relationship underscores the role of resilience as a protective factor, where personal competence acts as a buffer against stressors, helping students to sustain high academic performance [66]. This is supported by Wang et al. (2025), who identified modifiable resilience mechanisms—such as self-efficacy and cognitive flexibility—that mitigated stress among UK trainee teachers [67], and by Doney (2013), whose longitudinal case study highlighted how novice science teachers developed resilience through personal competencies and support systems to counteract professional stress and promote persistence [68].

According to Lazarus and Folkman’s Transactional Model of Stress and Coping, which highlights emotional regulation as a critical factor in stress management [49, 69]. Students with higher trust in one’s instincts and tolerance of negative effects (TITNA) may be more likely to interpret challenges constructively and use problem-focused coping strategies. This is supported by findings from Izquierdo et al. (2023), who found that emotional stability and positive emotional traits directly predicted mental health and adaptive academic functioning in trainee teachers, suggesting that emotional resilience components like TITNA are foundational for effective stress management in educational contexts [25]. In addition, Emotional resilience, as reflected in TITNA, further aligns with Broaden-and-Build Theory, which emphasizes how positive emotions like confidence broaden thought-action repertoires and build lasting personal resources [70].

Positive acceptance of change and secure relationships (PACSR)’s strong link to social adaptation could be explained by Bronfenbrenner’s Ecological Systems Theory, which emphasizes the role of relationships and adaptability in navigating social systems [71]. Students with higher PACSR leverage their adaptability and interpersonal skills to thrive socially within the academic context, fostering a sense of belonging and collaboration. This finding is consistent with the Resilience Theory [50], which highlights that adaptability and strong relationships act as external protective factors that enable individuals to thrive in adverse conditions. Empirical support for this could be seen in Diab and Green (2023), who found that pre-service teachers with higher resilience and optimism reported greater interpersonal satisfaction and well-being, underscoring how relational strengths and adaptability underpin effective social adjustment [72].

The absence of significant effects of resilience domains on study performance, regardless of cluster, highlights the complex and multifactorial nature of academic achievement. Academic performance is influenced by factors beyond individual psychological traits, such as external support (e.g., institutional resources and teaching quality), motivation, and cultural attitudes toward education [73–75]. According to Eccles and Wigfield’s Expectancy-Value Theory, academic success depends on a combination of individual expectancy (belief in one’s ability to succeed) and value (importance placed on success) [76]. While resilience supports adaptive functioning, its role may be indirect, mediated through factors such as motivation and goal setting. The low explanatory power (adjusted R² ≤ 0.01) further suggests that resilience alone is insufficient to predict performance outcomes directly.

Resilience’s role in coping with study demands varied across clusters, with differences emerging in the most influential domains. Among introverted individuals (Cluster 1), Control (CNT) was identified as a critical factor, emphasizing the value of self-regulation and decision-making when managing academic stress. This finding supports Lazarus and Folkman’s Transactional Model of Stress and Coping, which posits that individuals’ perception of control and ability to appraise stressors play a central role in determining adaptive responses. In the academic context, this model explains why students with higher control (CNT) perceive academic stress as manageable, thus fostering better coping strategies [69, 77]. On the other hand, Personal Competence, High Standards, and Tenacity (PCHST) emerged as critical for students facing emotional difficulties or those with extroverted profiles. This underscores the importance of self-discipline and perseverance in buffering emotional strain and maintaining academic function [65, 78].

When it came to social adaptation, Positive Acceptance of Change and Secure Relationships (PACSR) consistently emerged as the most influential resilience domain across all clusters. This dimension reflects students’ ability to adapt flexibly and form supportive interpersonal connections—factors shown to enhance social belonging and academic integration. Such findings echo prior research highlighting the role of peer and teacher support in facilitating emotional adjustment and relational resilience during college transitions [79, 80].

Finally, the absence of significant differences in model efficiency and structure across clusters suggests that resilience functions as a broadly applicable resource. According to Masten’s Ordinary Magic Theory, resilience is a universal human capacity that fosters adaptive functioning across diverse contexts [66]. Despite variations in the relative importance of specific resilience domains, their overall contribution to coping and social adaptation remained consistent, underscoring resilience’s role as a foundational psychological resource that transcends individual differences.

This study advances the understanding of resilience by highlighting its complex interaction with academic achievement, personality traits, and mental health. It confirms resilience as a universal psychological resource that transcends individual differences, aligning with Masten’s Ordinary Magic Theory (2014). The findings extend prior theories, such as Bronfenbrenner’s Ecological Systems Theory (1979), by demonstrating that adaptability and relational skills—captured by Positive Acceptance of Change and Secure Relationships (PACSR)—play critical roles in fostering social integration and academic outcomes across diverse student profiles. Furthermore, the use of cluster analysis to stratify participants provides new insights into the uniform applicability of resilience, emphasizing its socio-effective and dynamic dimensions in academic settings. These contributions enrich the broader discourse on resilience by situating its relevance within the educational and psychological contexts, particularly for future teachers.

The findings of this study offer profound implications for the education and support of future teachers. By identifying distinct resilience dimensions—such as Control (CNT), Personal Competence, High Standards, and Tenacity (PCHST), and Positive Acceptance of Change and Secure Relationships (PACSR)—differentially support coping and adaptation, this research provides a roadmap for targeted interventions in teacher education programs. Contrary to some earlier reports that resilience’s benefits might be dampened by high neuroticism or anxiety levels, our cluster-based analysis did not show such moderation. This could indicate that, when considering broader outcomes like coping and adaptation, resilience provides advantages across the board.

For pre-service teachers, who often face intense academic demands, professional identity development, and high emotional labor, resilience emerges as a trainable professional competency rather than a fixed trait. In this study, resilience primarily boosted coping with study demands and social adaptation, while showing only a minor, non-significant trend with grade-related performance. Accordingly, practical support should focus first on the psychosocial facets of academic success and then be paired with complementary study-skill or instructional supports when grade improvement is the goal.

For instance, introverted teacher candidates may benefit from workshops that promote autonomy, decision-making, and emotional self-regulation, thereby bolstering their classroom confidence and instructional clarity [81]. Meanwhile, candidates experiencing depressive or anxious symptoms may require structured support to cultivate perseverance, goal orientation, and self-efficacy—skills that are not only vital for academic endurance but also critical for managing classroom stress and student behavior once they enter the workforce. Previous study found that emotional exhaustion and psychological distress among teacher candidates were strongly linked to decreased professional engagement, underscoring the need for targeted interventions that enhance emotional resilience and self-regulatory skills in this population [82].

PACSR’s strong link to social adaptation further highlights the need to foster relational and adaptive skills in teacher education. These skills are fundamental to fostering inclusive classroom environments, maintaining positive peer and student-teacher relationships, and effectively navigating the interpersonal complexities of school communities [83]. Evidence-based practices—such as peer mentoring programs, BRiTE resilience modules, and collaborative reflection—have demonstrated significant benefits for pre-service teachers in enhancing emotional regulation, social confidence, and resilience during their training [84–86]. Embedding these interventions into teacher education curricula equips candidates with the tools to develop secure interpersonal bonds and adapt to evolving classroom dynamics, thereby laying the groundwork for both professional success and student engagement.

Moreover, by reinforcing the importance of resilience as a developmental and trainable attribute, this study advocates systemic integration of mental health resources in teacher preparation programs. This includes not only individual counseling and resilience training workshops but also pedagogical strategies that model and reinforce resilience-building behaviors—like growth mindset, reflective practice, and adaptive feedback. Ultimately, the study underscores that preparing resilient teachers is foundational to fostering resilient students, resilient schools, and a more sustainable educational system overall. Given the limited direct effect of resilience on grades, programmes should align resilience training with evidence-based study-skill instruction, formative assessment practices, and quality teaching methods to address grade outcomes more directly.

This study has several limitations that warrant attention. First, this study is cross-sectional; therefore, all findings are associative and do not establish temporal ordering or causality. Because all focal variables were collected via self-report at a single time point, potential common-method variance and social-desirability bias cannot be ruled out. Longitudinal studies would provide a more comprehensive understanding by exploring the temporal dynamics of these relationships and how resilience develops over time in response to academic and personal challenges.

Second, the sample was restricted to students in a teacher education program, which may constrain the generalizability of the findings to other academic disciplines, professional settings or even from different culture background. For example, the role of resilience in academic achievement appears to vary across different student populations, findings from Kvintová et al. (2024) revealed cross-cultural differences, with Czech students showing a more interconnected relationship between resilience, mental health, and academic achievement, whereas Chinese students exhibited more compartmentalized effects. These differences suggest that cultural and educational contexts shape how resilience interacts with academic outcomes [87]. Future research should include diverse student populations to determine whether the observed patterns extend to broader educational contexts.

Third, although cluster analysis yielded valuable insights into the interaction between resilience, personality, and mental health traits, the reliance on self-reported measures introduces potential biases, such as social desirability and inaccuracies in self-assessment. Incorporating objective assessments or multi-method approaches would strengthen the reliability of these findings. Lastly, the study primarily focused on resilience in relation to academic outcomes, potentially neglecting other critical aspects of student well-being, such as emotional health, motivation, and life satisfaction, which deserve further exploration.

Future research should aim to address these limitations and build on the insights generated by this study. Longitudinal designs could track the development of resilience over time, examining how its impact on academic outcomes and coping mechanisms shifts across different educational stages or life transitions. Expanding the participant pool to include students from various disciplines, cultural backgrounds, and educational systems would enhance the generalizability of findings and uncover potential cultural or contextual moderators of resilience. Additionally, future studies could explore how external factors, such as institutional support, family dynamics, or peer influence, interact with individual resilience domains to shape academic and social outcomes. To deepen understanding, researchers could also investigate the neurobiological or cognitive mechanisms underlying resilience, employing tools such as neuroimaging or experimental designs. Finally, integrating resilience into intervention studies would allow researchers to evaluate the efficacy of tailored programs and policies in improving both academic success and psychological well-being, providing actionable insights for educational and mental health practitioners.

## Conclusion

Resilience is significantly associated with aspects of academic achievement related to coping with study demands and social adaptation but is not directly associated with academic performance. This pattern is consistent across different student groups, regardless of their personality traits and mental health conditions. These findings underscore the potential importance of resilience as a crucial psychological resource associated with students’ ability to manage academic stress and integrate into their social and educational environment. Teacher training programs may benefit from integrating resilience-building strategies to potentially enhance student preparedness for academic and professional challenges. By fostering supportive learning environments and mental health awareness, institutions can contribute to a more resilient and effective future teaching workforce.

## Supplementary Information

Supplementary Material 1

Supplementary Material 2

Supplementary Material 3

## Data Availability

The datasets generated and/or analyzed during the current study are not publicly available due privacy and ethical restrictions about participants’ confidential information but are available from the corresponding author on reasonable request.

## Abbreviations

CD-RISC-25: Connor-Davidson Resilience Scale 25-item version
AAQ: Academic Achievement Questionnaire
TIPI: Ten Item Personality Inventory
PHQ-4: Patient Health Questionnaire 4-item version
PACSR: Positive Acceptance of Change and Secure Relationships
TITNA: Trust in One's Instincts, Tolerance of Negative Affect, and Strengthening Effects of Stress
PCHST: Personal Competence, High Standards, and Tenacity
CNT: Control
SI: Spiritual Influences
