# Supplementary material for: Resilience displays similar associative patterns with academic achievement regardless of the personality and mental health profile of future teachers
Source: BMC Psychol. 2025 Dec 11;14:163. doi: 10.1186/s40359-025-03697-7 (PMC12870039; doi:10.1186/s40359-025-03697-7)
Supplement: Supplementary file 3 — Supplementary Material 3 [file 40359_2025_3697_MOESM3_ESM.pdf]

Vážení,

tímto dokumentem potvrzuji, že Katedra psychologie Metropolitní univerzity Praha výzkumně spolupracuje s Katedrou psychologie a patopsychologie PedF UP Olomouc a v této souvislosti používá Czech Connor-Davidson Resilience Scale (CD-RISC), námi zakoupenou metodu od Jonathan R. T. Davidson, M.D.

S pozdravem,

PhDr. Markéta Niederlová, Ph.D.
vedoucí katedry psychologie
Metropolitní univerzita Praha

METROPOLITNÍ UNIVERZITA PRAHA, o.p.s.
Sídlo: Dubečská 900/10, 100 00 Praha 10
(4)
