# Supplementary material for: Resilience displays similar associative patterns with academic achievement regardless of the personality and mental health profile of future teachers
Source: BMC Psychol. 2025 Dec 11;14:163. doi: 10.1186/s40359-025-03697-7 (PMC12870039; doi:10.1186/s40359-025-03697-7)
Supplement: Supplementary file 2 — Supplementary Material 2 [file 40359_2025_3697_MOESM2_ESM.pdf]

Pedagogická
fakulta

V Olomouci dne 3. 3. 2025

Vážená paní
PhDr. Jana Kvintová, Ph.D.
Katedra psychologie a patopsychologie

Stanovisko Etické komise pro oblast vědy a výzkumu Pedagogické fakulty Univerzity Palackého v Olomouci

Vážená paní doktorko,

Etická komise pro oblast vědy a výzkumu Pedagogické fakulty Univerzity Palackého v Olomouci, zřízená dle vnitřní normy PdF UP PdF-B-23/07 *Etická komise pro oblast vědy a výzkumu* a jmenovaná ve složení
prof. PaedDr. Milan Valenta, Ph.D.,
doc. PhDr. Irena Plevová, Ph.D.,
doc. PhDr. Vlasta Řeřichová, CSc.,
PhDr. Ivona Dömischová, Ph.D.,
doc. Mgr. et Mgr. Jan Chrastina, Ph.D., LL.M., MBA,
projednala Vaši žádost o posouzení etických aspektů výzkumného projektu *Odolnost a akademický úspěch budoucích učitelů: Modelující role osobnostních rysů a duševního zdraví* a usnesla se, že předložený projekt podmínky etické přípustnosti realizace výzkumu

splňuje.

Přeji Vám mnoho úspěchů ve Vaší práci.

prof. PaedDr. Milan Valenta, Ph.D.
Předseda Etické komise pro oblast vědy a výzkumu PdF UP

Pedagogická fakulta Univerzity Palackého v Olomouci
Žižkovo nám. 5 | 771 40 Olomouc | T: 585 635 099
www.pdf.upol.cz

Olomouc, dated 3. 3. 2025

Dear madam
PhDr. Jana Kvintová, Ph.D.
Department of Psychology and Psychopathology

Statement of the Ethics Commission for Science and Research of the Faculty of Education, Palacký University Olomouc

Dear dr. Kvintová,

The Ethics Commission for Science and Research of the Faculty of Education, Palacký University Olomouc, established pursuant to the Internal Norm of PdF UP PdF-B-23/07 Ethics Commission for Science and Research with the following appointed members
Prof. PaedDr. Milan Valenta, Ph.D.,
doc. PhDr. Irena Plevová, Ph.D.,
doc. PhDr. Vlasta Řeřichová, CSc.,
PhDr. Ivona Dömischová, Ph.D.,
doc. Mgr. et Mgr. Jan Chrastina, Ph.D., LL.M., MBA,
has considered your request for an assessment of the ethical aspects of the research *Resilience and Academic Achievement in Future Teachers: The Moderating Role of Personality Traits and Mental Health* and has decided as follows:

The project **meets** the conditions of ethical admissibility in research.

I wish you every success in your work.

Prof. PaedDr. Milan Valenta, Ph.D.
Chairperson of the Ethics Commission for Science and Research of the Faculty of Education UP
